# Deconjugated Bile Salts Produced by Extracellular Bile-Salt Hydrolase-Like Activities from the Probiotic *Lactobacillus johnsonii* La1 Inhibit *Giardia duodenalis In vitro* Growth

**DOI:** 10.3389/fmicb.2016.01453

**Published:** 2016-09-27

**Authors:** Marie-Agnès Travers, Cissé Sow, Séverine Zirah, Christiane Deregnaucourt, Soraya Chaouch, Rayner M. L. Queiroz, Sébastien Charneau, Thibault Allain, Isabelle Florent, Philippe Grellier

**Affiliations:** ^1^Laboratoire de Génétique et Pathologie des Mollusques Marins, Unité SG2M, IFREMERLa Tremblade, France; ^2^MCAM UMR 7245, Muséum National d'Histoire Naturelle, Centre National de la Recherche Scientifique, Sorbonne UniversitésParis, France; ^3^Department of Cell Biology, Institute of Biology, University of BrasiliaBrasília, Brazil; ^4^UMR 1319, Commensal and Probiotics-Host Interactions Laboratory, INRA, AgroParisTechJouy en Josas, France

**Keywords:** giardiasis, lactobacilli, probiotics, microbiota, bile salts, anti-giardial activity, choloylglycine bile acid hydrolase

## Abstract

Giardiasis, currently considered a neglected disease, is caused by the intestinal protozoan parasite *Giardia duodenalis* and is widely spread in human as well as domestic and wild animals. The lack of appropriate medications and the spread of resistant parasite strains urgently call for the development of novel therapeutic strategies. Host microbiota or certain probiotic strains have the capacity to provide some protection against giardiasis. By combining biological and biochemical approaches, we have been able to decipher a molecular mechanism used by the probiotic strain *Lactobacillus johnsonii* La1 to prevent *Giardia* growth *in vitro*. We provide evidence that the supernatant of this strain contains active principle(s) not directly toxic to *Giardia* but able to convert non-toxic components of bile into components highly toxic to *Giardia*. By using bile acid profiling, these components were identified as deconjugated bile-salts. A bacterial bile-salt-hydrolase of commercial origin was able to mimic the properties of the supernatant. Mass spectrometric analysis of the bacterial supernatant identified two of the three bile-salt-hydrolases encoded in the genome of this probiotic strain. These observations document a possible mechanism by which *L. johnsonii* La1, by secreting, or releasing BSH-like activity(ies) in the vicinity of replicating *Giardia* in an environment where bile is present and abundant, can fight this parasite. This discovery has both fundamental and applied outcomes to fight giardiasis, based on local delivery of deconjugated bile salts, enzyme deconjugation of bile components, or natural or recombinant probiotic strains that secrete or release such deconjugating activities in a compartment where both bile salts and *Giardia* are present.

## Introduction

*Giardia duodenalis* is a parasitic protozoa responsible for giardiasis, a disease characterized by acute or chronic intestinal malabsorption, diarrhea, weight loss, dehydration, and abdominal pain in humans and a variety of vertebrates. It is one of the most common intestinal parasites, with 200–300 million human cases estimated worldwide per year (Lane and Lloyd, 2002). Giardiasis has high veterinary impact and high impact on public health, and is responsible for human morbidity, especially causing nutritional deficiencies in children in developing countries (Lane and Lloyd, 2002; Ali and Hill, 2003; Veenemans et al., 2011). Developed countries are also concerned by giardiasis; outbreaks have been associated with drinking water contamination resulting from runoff of contaminated soils by rain falls, agricultural practices, and sewage treatment plant dysfunctions (Mons et al., 2009; Baldursson and Karanis, 2011).

*Giardia duodenalis* exists under two development forms, a resistant form called cyst, responsible for the transmission of the parasite between vertebrate hosts, and an active form called trophozoite, that replicates within intestinal tracts of hosts. Cysts enter vertebrate hosts *via* several routes such as food, water, or fomites contaminated by feces from infected hosts. Cysts remain infective for months in environmental waters and are infectious at low doses (10–100 cysts; Lane and Lloyd, 2002). Upon parasite excystation, newly formed trophozoites are released and colonize the surface of the intestinal barrier in the upper small intestine (duodenum). The duodenum constitutes a very specific microenvironment where chyme from the stomach, bile produced from the liver and stored in the gall bladder, and digestive enzymes from the pancreas pour in. After encystation in the lower part of the small intestine, parasites exit the host in the feces (Lane and Lloyd, 2002; Ali and Hill, 2003; Ankarklev et al., 2010).

The pathophysiology of giardiasis is multifactorial and comprises damages to the host's mucosal surface such as diffuse microvillous shortening and a reduction of villus-crypt ratios. An increase of epithelial permeability, which might lead to a bacterial translocation is also observed as well as an impairment of disacharridase activity of digestive enzymes (Cotton et al., 2011; Buret et al., 2015). These changes may be due as much to factors of the host as to those of the parasite. In recent studies, a post-infectious link has been established between *Giardia* infection and intestinal- and extra-intestinal disorders such as inflammatory bowel syndrome and chronic fatigue syndrome (Halliez and Buret, 2013; Ventura et al., 2004). The drug of choice for treating giardiasis remains metronidazole, a 5-nitroimidazole (Leitsch et al., 2011). However, drug side effects (vertigo, nausea, vomiting, anorexia, and dizziness; Gardner and Hill, 2001) and the occurrence of drug resistant strains (Upcroft and Upcroft, 2001; Lalle, 2010; Tian et al., 2010; Ansell et al., 2015) make research on alternative therapeutic strategies necessary.

In this context, it is now widely recognized that the intestinal microbiota plays a role in the protection of the host against the gut colonization by pathogens (Singer and Nash, 2000; Travers et al., 2011; Kamada et al., 2013; Bartelt and Sartor, 2015). Different mechanisms may be responsible for these protective effects such as competition for pathogen ecological niches, competition for nutritional substrates, production of antimicrobial compounds, and enhancement of the innate and adaptive host immune responses (Tancrède, 1992; Kamada et al., 2013). Works highlighted the importance of intestinal bacteria in *G. duodenalis* infection (Singer and Nash, 2000). Evidence suggests that the bacterium *Enterococcus faecium* contributes to the clearance of *G. duodenalis* by enhancing the host immune response (Benyacoub et al., 2005). Additionally, the probiotic bacterium *Lactobacillus johnsonii* La1 antagonizes *G. duodenalis* establishment in rodent models (Humen et al., 2005) and extracellular factors of *L. johnsonii* La1 arrest the *in vitro* growth of *G. duodenalis* in the G1 phase indicating that this bacterium may directly affect parasite replication (Pérez et al., 2001). A better knowledge of the molecular interactions between *G. duodenalis* and the duodenal microbiota could thus open new chemotherapeutic strategies based on the inhibition of specific parasite enzymes or processes. *L. johnsonii* La1 constitutes a model of choice to study the molecular crosstalks between *G. duodenalis* and probiotic bacteria such as lactobacilli, which are one of the most common bacteria in the duodenum. Anti-*Giardia* activity was clearly demonstrated *in vivo* and *in vitro* (Pérez et al., 2001; Humen et al., 2005) and the genomes of *L. johnsonii* La1 and *G. duodenalis* have been sequenced (Pridmore et al., 2004; Morrison et al., 2007; Franzén et al., 2009).

In the present paper, we investigated the molecular mechanisms involved in the *in vitro* anti-*Giardia* activity of extracellular factors of the probiotic *L. johnsonii* La1. *G. duodenalis* can be cultured *in vitro* (Keister, 1983). Bile was historically added into *Giardia* culture medium to promote its *in vitro* growth, even if this gain in generation time could be reduced at high bile and bile salt concentrations (Farthing et al., 1983, 1985). Parasites can internalize bile lipids as metabolites or source of phospholipids for membrane biosynthesis (Farthing et al., 1985; Halliday et al., 1995; Das et al., 1997). Conjugated bile salts have also a role in promoting encystation (Gillin, 1987; Gillin et al., 1989). We found that the parasite growth inhibition by *L. johnsonii* La1 culture spent supernatant is mediated by secreted bacteria bile-salt hydrolase-like activity(ies) that generate(s) deconjugated bile salts from bile present in the culture medium. Unlike conjugated bile salts, the main components of bile, deconjugated bile salts were found to be toxic to *Giardia*.

## Materials and methods

### Products and preparation of stock solutions

Bovine bile (reference B3883, Sigma-Aldrich and reference 212820, Difco, BD Diagnostic Systems) was prepared at 100 mg/ml stock solution in distilled water, sterilized through 0.2 μm filters and stored at −20°C. Pure bile salts: glycocholate (GC), taurocholate (TC), glycodeoxycholate (GDC), taurodeoxycholate (TDC), glycochenodeoxycholate (GCDC), and taurochenodeoxycholate (TCDC), bile salt mix or pure deconjugated bile salts: cholate (C), deoxycholate (DC), and chenodeoxycholate (CDC), and fusidic acid were from Sigma-Aldrich and dissolved in distilled water to 12 mg/ml stock solutions, filtered through 0.2 μm pores and kept at −20°C. Choloylglycine Bile Acid Hydrolase or Bile Salt Hydrolase (BSH, EC 3.5.1.24) from *Clostridium perfringens* (reference C4018, Sigma-Aldrich) was prepared at 10 U/ml in distilled water and stored at −20°C. Fetal calf serum (FCS, reference A15-101, PAA Laboratories, GE Healthcare), and bovine calf serum (reference B9433, Sigma-Aldrich) were used. The NEFA-C kit used for quantitative determination of non-esterified fatty acids (NEFAs) was from Biolab, WAKO Diagnostics.

### Cell culture of *Giardia duodenalis*

Trophozoites of *G. duodenalis* strains WB (clone C6, ATCC 30957) and HP1 (Portland-1, gift of Jan Tackezy, Charles University of Prague, Czech Republic) from assemblage A were routinely maintained in axenic culture in TYI-S-33 Keister's medium (KM) as previously described (Keister, 1983). Parasites were grown in 15 ml tubes filled with 12 ml culture medium. They were regularly subcultured at a density of 5 × 10^4^ cells per tube (12 ml) from log phase parasites chilled on ice for 10 min and centrifuged at 700 × *g*, 5 min. For bioassays, parasites were maintained in culture in KM adjusted to pH 6.0, supplemented with 10% heat-inactivated FCS (Paget et al., 2004) and 0.6 g/L bovine bile (Carnaby et al., 1994).

### Culture of *Lactobacillus johnsonii* La1 and production of bacterial supernatant

*L. johnsonii* La1 (CNRZ 1897, NCC533) was kindly provided by Pascal Quénée (INRA Jouy en Josas, Equipe Atalis, France) and was isolated from LC1 product in 1996 (Chambourcy, France). The presence of *L. johnsonii* La1 prophage, as previously described (Ventura et al., 2004; Denou et al., 2008), was checked by PCR using two primer pairs: 1DF/1B2R resulting in a 1500 bp amplicon for *L. johnsonii* “La1-like” (with the prophage) and 1EF/1B2R resulting in a 268 bp amplicon as a positive control for *L. johnsonii* strains. PCR reactions were run in a final volume of 25 μl containing 200 ng of bacterial DNA extracted with a standard protocol (Zhong et al., 1998), 200 nM of each primer (1DF: AGA CTT TTT GGC AGG CAA AGG, 1EF: ACA AAC CAC CAG TGC CTA AGG, 1B2R: GCT CTT CGA GAT CAC TGG GC), 200 nM of dNTP, and 1U of Taq DNA polymerase (NEB M0273S). Reactions were initiated with an initial denaturation for 5 min at 95°C followed by 35 cycles at 95°C for 30 s, 50°C for 30 s and 72°C for 1 min. PCR products were visualized on 0.8% agarose gel containing 0.5 ng/μl of BET and images were captured with a camera coupled with Biocapt software, Vilber Lourmat.

*L. johnsonii* La1 stock cultures were kept frozen in de Man Rogosa Sharpe (MRS) Broth (Sigma-Aldrich) media with 15% glycerol. Bacteria were subcultured in MRS or modified TYI-S-33 medium (MTYI) (Pérez et al., 2001) and incubated anaerobically for 12–18 h at 37°C. Bacteria were subsequently grown in MTYI or KM supplemented with or without 10% heat-inactivated FCS for 12–16 h in the presence or the absence of 0.6 g/L bovine bile. After centrifugation (3000 × *g*, 10 min) and 0.2 μm filtration, the pH of the *L. johnsonii* La1 supernatant was adjusted to 6.0 with 5 N NaOH. For some experiments, pH was adjusted to 6.2, 6.7, 6.9, or 7.2. Appropriated controls were prepared as follows: lactic acid produced during growth was quantified from aliquots of supernatants (Enzytec™ kit, R-Biopharm) and an equivalent amount of lactic acid was added to fresh medium before pH adjustment.

### *In vitro G. duodenalis* growth inhibition assay

One milliliter of trophozoite suspension (1 × 10^5^ parasites/ml in KM, pH 6, supplemented with 10% FCS without bovine bile) was mixed with either 500 μl of *L. johnsonii* La1 supernatant or one, or two, units of commercial bile salt hydrolase (BSH) from *C. perfringens*, chromatography purified fractions or appropriate control medium. These suspensions were further mixed with different concentrations of either bovine bile, mixed bile salts, conjugated, or deconjugated bile salts or appropriate control medium. Samples were then incubated for 24 h at 37°C and chilled on ice for 10 min to dislodge trophozoites from tube wall. Trophozoites were counted using a Malassez cell chamber and were considered alive when parasites showed the typical pear shape and signs of flagella mobility (Video S1). Morphologically altered parasites with non-mobile flagella were considered “dead” (Video S2). Estimates of parasite viability on these criteria correlate with parasite viability measured by propidium iodide staining and flow cytometry analysis (Barbosa et al., 2008; Figure S1). Multiplication factor [i.e., number of total trophozoites at the end of the experiment/number of trophozoites at time zero], survival rate [i.e., (number of living cells/total number of trophozoites) × 100], and inhibition percentage [i.e., (100-number of living cells in the presence of tested compounds/number of living cells in control) × 100] were calculated.

### Partial purification of active fractions from *L. johnsonii* La1 supernatants by gel filtration

Supernatants from *L. johnsonii* La1 cultures in KM, adjusted to pH 6.0, were concentrated up to 30-fold by ultrafiltration using the 10 kDa Centriprep centrifugal filter unit (Millipore). After 0.2 μm filtration, the concentrated supernatants were loaded onto a Sephacryl S300 column 16/100 (GE Healthcare), previously equilibrated with 20 mM ammonium sulfate (pH 6.0), in a cold room and were eluted with the same buffer at a flow rate of 2.0 ml/min. Effluent fractions of 12 ml were collected, concentrated 4-fold by ultrafiltration on a 10 kDa Centriprep and tested for *in vitro Giardia* growth inhibition in the presence of bovine bile or bile salts. Fractions obtained by similar processing of elution buffer alone and control media containing lactic acid (see above) were used as controls. Column calibration was carried out with ribonuclease A (13.7 kDa) and bovine serum albumin (67 kDa).

### Characterization of the inhibitory molecule(s) in *L. johnsonii* La1 supernatant

The molecular size of inhibitory molecule(s) present in *L. johnsonii* La1 supernatant was assessed by ultrafiltration using 10, 30, and 50 kDa Centriprep centrifugal filter units. Thermal stability was tested by heating the bacterial supernatant to 90°C for 10 min. Preservation of *L. johnsonii* La1 supernatant activity upon dialysis was checked by dialyzing twice (at 4°C for 2 and 15 h, respectively) the supernatant against 100 volumes of KM supplemented with 10% FCS or against GKN solution (NaCl, 8 g/l; KCl, 0.4 g/l; glucose, 2 g/l; NaH_2_PO_4_, H_2_O, 0.69 g/l; Na_2_HPO_4_, 1.57 g/l; pH 7.2–7.4) (Pérez et al., 2001) using a dialysis membrane with a molecular weight cut-off of 3.5 kDa (Spectrum Laboratories). The dialyzed supernatant was then filtrated through a 0.2 μm membrane and kept frozen at −80°C prior to *G. duodenalis* inhibition assays.

The biochemical nature of inhibitory molecule(s) was investigated by preincubating the 5-fold concentrated *L. johnsonii* La1 supernatant obtained by ultrafiltration (>10 kDa) with different enzymes coupled to beads. Briefly, proteinase K (Invitrogen), pronase and catalase (Merck) were coupled to CNBr-activated Sepharose™ 4B beads (GE Healthcare) following manufacturer's instructions. Five milliliters of the 5-fold concentrated *L. johnsonii* La1 supernatant or the 5-fold concentrated fresh control medium were incubated for 6 h at room temperature (RT) in the presence of 100 μl of packed enzyme-coupled beads. Beads were removed by centrifugation (4000 × *g*, 5 min) before parasite growth inhibition assays.

### Measurement of free fatty acids

To assess the presence of free fatty acids in the complex medium inducing *G. duodenalis* growth inhibition, FCS, bile, and *L. johnsonii* La1 supernatant were analyzed for non-esterified fatty acid (NEFA) content either alone or in combination in KM. Respective concentrations of the components in samples were as follows: FCS 10% (v/v), bile 0.6 g/L and *L. johnsonii* La1 supernatant 33.3% (v/v) final concentration in KM, pH 6. Samples (0.5 ml) were kept on ice before being incubated for 24 h at 37°C in the presence of 4.8 × 10^4^ trophozoites or without parasites. At the end of the incubation period, tubes were chilled on ice, centrifuged at 700 × *g*, 10 min at RT. The supernatants were then taken and frozen at −80°C before NEFA measurement. Numbers of living and “dead” trophozoites (as defined above) were determined using a Malassez cell chamber. NEFAs were quantified using the spectrometric NEFA-C kit method, based on Duncombe (1964) and Itaya and Ui (1965) studies, following manufacturer's instructions. Oleic acid was used as a standard and NEFAs were expressed as oleic acid equivalents (Eq).

### Bile salt hydrolase (BSH) activity assays of chromatography fractions

After gel filtration, the eluted fractions were concentrated 10-fold by dialysis against 20 mM ammonium acetate buffer containing 2 M sucrose, pH 6.0, using a 3.5 kDa molecular weight cut-off membrane (Spectrum Laboratories). Bile salt GDC was used for enzymatic assays. BSH activity was monitored by measuring glycine liberation from conjugated bile salts following the protocol described by Grill et al. (2000). Briefly 100 μl of effluent fractions or 1 unit of BSH or elution buffer were mixed with 100 μl of 2.4 g/l of GDC and incubated for 24 h at 37°C. Controls were performed in the absence of the bile salt. The enzymatic reaction was stopped by addition of an equal volume of 15% TCA. Precipitated proteins were pelleted by centrifugation at 20,000 × *g* for 15 min and the supernatant was carefully removed. To 80 μl of supernatant, 680 μl of 0.3 M borate buffer, 1% SDS, pH 9.5 and 80 μl of 0.3% picrylsulfonic acid solution (Sigma-Aldrich) were added. The mixture was incubated for 30 min in the dark and 800 μl of 1 mM HCl was added to stop the reaction. Glycine concentration was measured at 416 nm using an Uvikon spectrophotometer 930 (Kontron Instruments). A standard curve was established using free glycine (Sigma-Aldrich).

### LC/ESI-MS analysis of modifications of bile components by *L. johnsonii* La1 supernatants

*L. johnsonii* La1 supernatant prepared in KM, pH 6.0, supplemented with 10% FCS and 1 g/L bovine bile was incubated overnight at 37°C. Two different bile batches and two different *L. johnsonii* La1 supernatant preparations were tested. Controls constituted of KM alone and heat-treated *L. johnsonii* La1 supernatant (90°C, 10 min). The sample preparation and LC-MS protocol were adapted from the conditions reported in the literature on the LC-MS analysis of bile (Mitamura et al., 2011) and bile acids (Perwaiz et al., 2001; Griffiths and Sjövall, 2010). After incubation, the samples were diluted 4-fold in Milli-Q water and subjected to solid-phase extraction using Oasis® HLB cartridges (30 mg solid phase). After conditioning with 3 ml methanol and 3 ml Milli-Q water, the cartridges were loaded with 1 ml of the 4-fold diluted samples, washed with 2 ml Milli-Q water and eluted with 2 ml methanol. The eluted fractions were vacuum dried and resuspended in 500 μl Milli-Q water/acetonitrile 90:10 (v/v). 5 μl of each resuspended sample was analyzed by liquid chromatography coupled electrospray ionization-mass spectrometry (LC/ESI-MS) on an Ultimate U3000 chromatographic system (Thermo Scientific) connected to a Q-STAR Pulsar Qq-TOF mass spectrometer equipped with an ionspray source (Sciex). The LC separation was achieved by an Interchrom Strategy C18-2 micro column (5 μm, 150 × 1 mm, 100 Å, Interchim). The elution gradient was 10% mobile phase B (acetonitrile) to 70% B against mobile phase A (5 mM ammonium formate/formic acid, pH 6) over 45 min, at a flow rate of 40 μl/min. The MS data were acquired in negative mode in the range *m/z* 250–1200. Each LC/ESI-MS experiment was conducted twice. Data-dependent LC/ESI-MS/MS experiments were also conducted on each sample, alternating 1-s full-scan MS followed by 2-s product ion collision induced dissociation of the major ions detected at the first step, using a −50 V collision voltage.

The raw LC/ESI-MS data were converted into Network Commun Data Form (NetCDF) using the translation tool provided by Sciex and processed with the XCMS package (Smith et al., 2006), a software implemented in the freely available R environment (www.r-project.org). This analysis allows automatic retention time alignment, matched filtration, peak detection, and peak matching.

### Proteomic analysis of *L. johnsonii* La1 supernatant

Sodium lauroyl sarcosinate (NLS, 0.1%, Sigma-Aldrich) was added to *L. johnsonii* La1 supernatant. After mixing, trichloroacetic acid (TCA) was added to a final 7.5% concentration and the solution was precipitated on ice overnight. Mixed protein-detergent precipitate was collected by centrifugation (10,000 × *g*, 10 min, 4°C). The supernatant was carefully removed and the pellet was washed twice with 2 ml of precooled tetrahydrofuran (Sigma-Aldrich). The pellet was then resuspended in 8 M urea in 20 mM triethylammonium bicarbonate and incubated for 1 h at 20°C with 20 mM DTT, followed by incubation with 50 mM iodacetamide for 1 h at 20°C in the dark. The sample was incubated with 0.05 U of endoproteinase Lys-C (Wako Pure Chemical Industries) for 18 h at RT. Then, the samples were diluted with 20 mM triethylammonium bicarbonate to a final concentration of 1 M urea and trypsin (Promega) digestion was performed with 2 μg of enzyme for 4 h at 20°C and terminated with a final concentration of 0.5% trifluoroacetic acid. The sample was passed sequentially through two home-made Poros Oligo-R3 (PerSeptive Biosystems) microcolumns packed (~1 cm) on p200 tips over 3MM C18 material plug. Loaded resin was washed with 100 μl 0.1% trifluoroacetic and peptides were eluted with 100 μl 70% acetonitrile/ 0.1% trifluoroacetic, then 20 μl 100% acetonitrile. The desalted sample was dried down, resuspended in 50% acetonitrile, dried, and stored at −80°C. Part of the sample (10%) was collected for amino acid analysis using a Biochrom 30 amino acid analyzer.

Samples (3 μg per run) were analyzed by an EASY-nano LC system (Proxeon Biosystems) coupled online to an LTQ-Orbitrap Velos mass spectrometer (Thermo Scientific). Peptides were loaded onto a 18 cm fused silica emitter (75 μm inner diameter) packed in-house with reverse phase capillary column ReproSil-Pur C18-AQ 3 μm resin (Dr. Maisch GmbH) and eluted using a gradient from 100% phase A (0.1% formic acid) to 35% phase B (0.1 formic acid, 95% acetonitrile) for 180 min, 35 to 100% phase B for 5 min and 100% phase B for 8 min (a total of 23 min at 250 nl/min). After each run, the column was washed with 90% phase B and re-equilibrated with phase A. Mass spectra were acquired in positive mode applying data-dependent automatic survey MS scan and tandem mass spectra (MS/MS) acquisition. Each MS scan in the orbitrap (mass range of m/z of 400–1800 and resolution 100,000) was followed by MS/MS of the 15 most intense ions in the LTQ. Fragmentation in the LTQ was performed by collision-induced dissociation and selected sequenced ions were dynamically excluded for 25 s.

Raw data were viewed in Xcalibur v.2.1 (Thermo Scientific) and data processing was performed using Proteome Discoverer v.1.3 (Thermo Scientific). The generated raw files were submitted to searching using Proteome Discoverer with an in house Mascot v.2.3 algorithm against a database of proteins predicted from the *L. johnsonii* isolates NCC533 and FI9785 and the *L. johnsonii* prophage Lj965 (http://www.ncbi.nlm.nih.gov/bioproject). Contaminant proteins (human keratins, BSA and porcine trypsin) were also added to the database and all contaminant proteins identified were manually removed from the result lists. The searches were performed with the following parameters: ms accuracy 10 ppm, MS/MS accuracy 0.6 Da, trypsin digestion with one missed cleavage allowed, fixed carbamidomethyl modification of cysteine and variable modification of oxidized methionine and N-terminal protein acetylation. The number of proteins, protein groups, and number of peptides were filtered for False Discovery Rates (FDR) less than 1%. Only peptides with rank 1 and minimal of 2 peptides per protein were accepted for identification using Proteome Discoverer (Charneau et al., 2007; Queiroz et al., 2013).

### Statistical analyses

Significant differences between treatments were tested using the “Kruskal-Wallis” test in http://marne.u707.jussieu.fr/biostatgv/. Results were considered significant at *P* < 0.05.

## Results

### The anti-*Giardia* growth effect, mediated by *L. johnsonii* La1 supernatant, is dependent upon bovine bile and more specifically upon bile salts

Pérez et al. (2001) showed that the inhibitory effect of *L. johnsonii* La1 supernatant upon *in vitro G. duodenalis* growth was pH dependent with a more pronounced inhibitory effect at pH 6 than pH 7. Change of culture medium pH had a slight effect on parasite proliferation and adhesion properties. We thus set up our culture assays at pH 6. FCS was used instead of adult bovine serum for better reproducibility of the assay. In our experimental conditions, parasite morphology, motility, and viability measured by propidium iodide staining were unaffected (Video S1, Figure S1).

**Figure 1:**
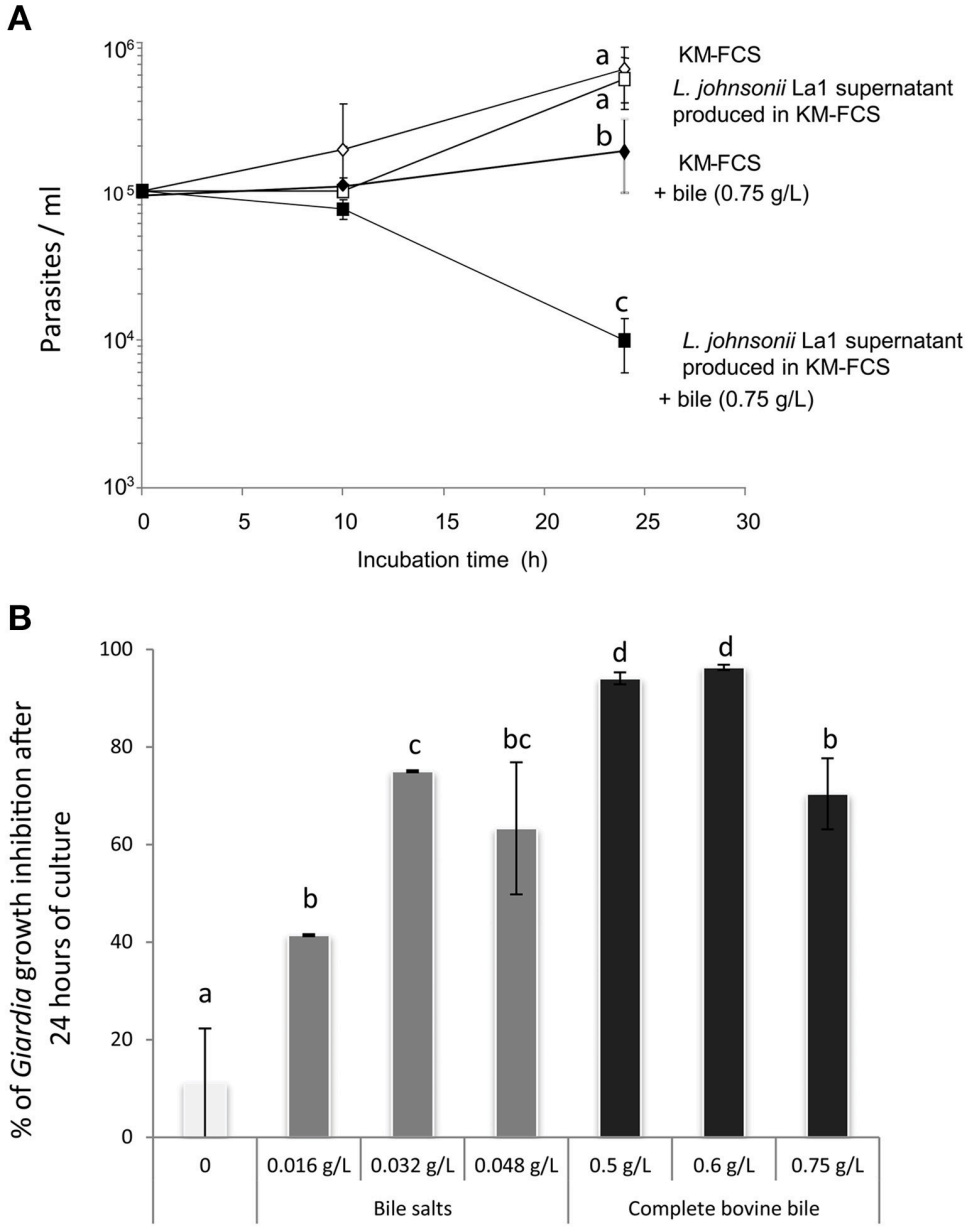
**(A)** The inhibitory effect of *L. johnsonii* La1 supernatant on *G. duodenalis* growth is observed after 24 h in the presence of bovine bile. *G. duodenalis* trophozoites were grown in KM-FCS with bovine bile (0.75 g/L, final concentration) in the presence (■) or in the absence (♦) of bacterial supernatant, or without bovine bile in the presence (□) or in the absence (◊) of bacterial supernatant. The parasite concentration was estimated by counting live cells with a Malassez cell chamber. Values are the mean ± SD of two independent experiments performed in triplicate. Letters indicate significant differences between treatments (Kruskall-Wallis, *p* < 0.05). **(B)** *G. duodenalis* growth inhibition by *L. johnsonii* La1 supernatant depends on the presence of bile, more specifically of bile salts. *G. duodenalis* trophozoites in KM-FCS were incubated for 24 h with *L. johnsonii* La1 supernatant and various concentrations of mixed bile salts (0.016, 0.032, 0.048 g/L, final concentration) or complete bovine bile (0, 0.5, 0.6, 0.75 g/L, final concentration). Growth inhibition values (%) were normalized according to controls in lactic acid-acidified KM-FCS supplemented with similar concentrations of bovine bile or mixed bile salts. Values are the mean ± SD of three independent experiments. Letters indicate significant differences between treatments (Kruskall-Wallis, *p* < 0.05).

Most media previously described to support *G. duodenalis* growth *in vitro* commonly contains bile as a supply for parasite cholesterol and fatty acid requirements (Farthing et al., 1985; Gillin et al., 1986; Halliday et al., 1995). In our hands, *G. duodenalis* trophozoites growth was observed in the absence of bovine bile. Bile, at the recommended concentration (0.75 g/L) (Pérez et al., 2001), appeared to reduce trophozoite proliferation when added to KM-FCS (Figure 1A). We next confirmed the *in vitro* inhibitory effect of *L. johnsonii* La1 supernatant upon *G. duodenalis* growth. Interestingly, the inhibitory effect was only observed in the presence of bovine bile. No inhibitory effect was observed in the absence of bovine bile, even after 24 h of culture (Figure 1A). After 10 h in the presence of 0.75 g/L of bovine bile, *G. duodenalis* trophozoite survival was slightly impacted by *L. johnsonii* La1 supernatant, but survival was largely affected after 24 h of contact (9.10^4^ and 1.10^4^ trophozoite/ml, respectively, Figure 1A, Video S2, Figure S1). Thus, a 24 h incubation time-period was retained for all subsequent inhibitory growth assays.

Maximal inhibitory effects of *L. johnsonii* La1 supernatant on parasite growth were observed either in the presence of 0.6 g/L of complete bovine bile or in the presence of 0.032 g/L of bile salt mix (96.3 and 75% of inhibition, respectively, by comparison with control media containing the same amount of lactic acid, bile or bile salt mix, Figure 1B, *p* < 0.05). Similar inhibitory effects were observed using two isolates of *G. duodenalis* (WB and HP1), two different commercial origins of complete bovine bile (Figure S2) and two different media compatible with bacteria and parasite growth: MTYI (Pérez et al., 2001) and KM-FCS (Paget et al., 2004). Unless otherwise indicated, subsequent *in vitro G. duodenalis* inhibition growth assays were performed using KM as medium at pH 6 and 0.6 g/L of complete bovine bile.

### Biochemical characterization of inhibitory activity

#### *L. johnsonii* extracellular products inhibiting *Giardia* growth are of peptidic nature

**Figure 2:**
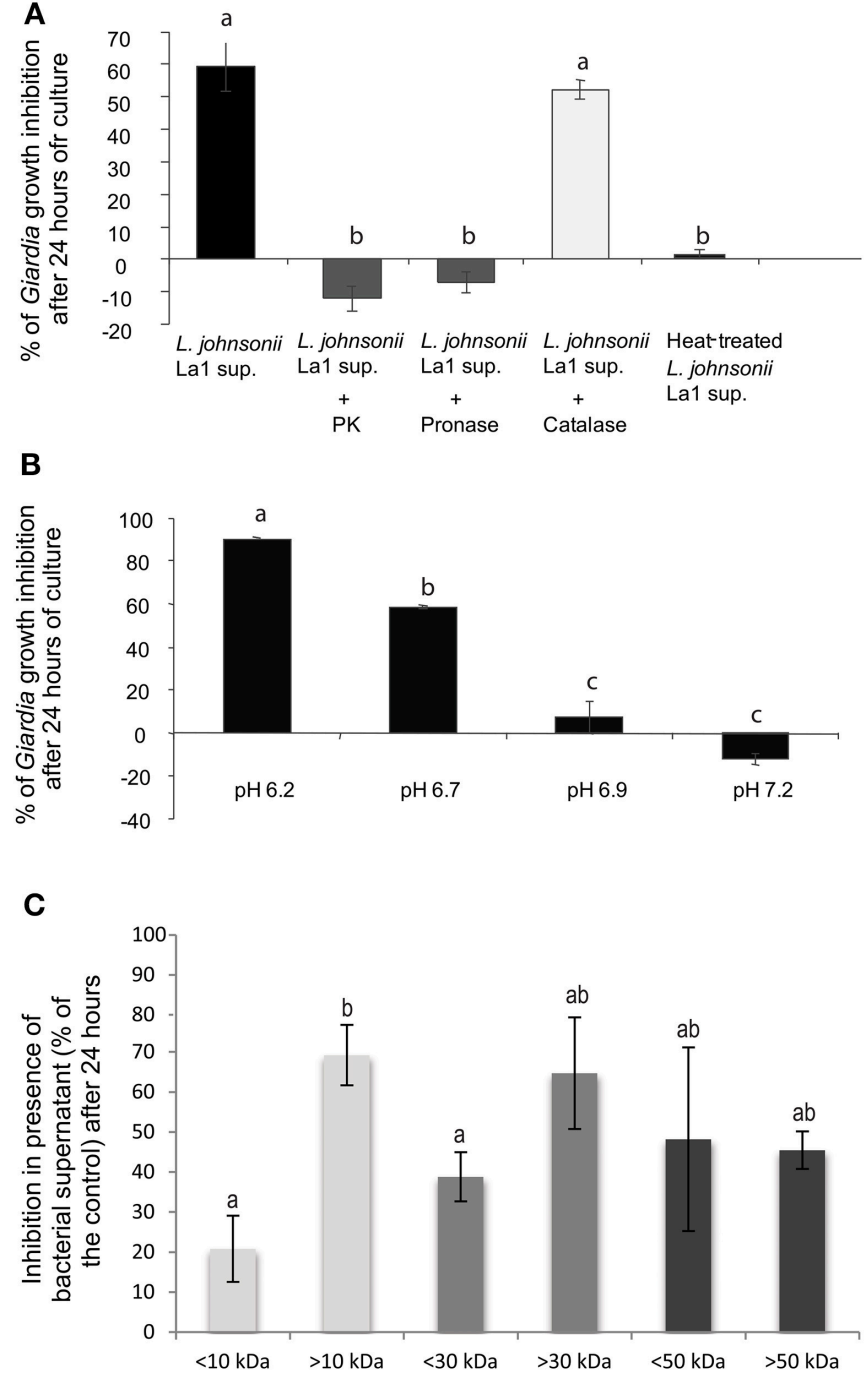
***G. duodenalis* growth inhibition by *L. johnsonii* La1 supernatant is affected by supernatant incubation with proteases, heat-treatment and pH. (A)** Supernatant of *L. johnsonii* La1 was incubated for 6 h with 1 mg of immobilized proteases (*L. johnsonii* La1 supernatant: no protease treatment; *L. johnsonii* La1 supernatant + PK: treatment with proteinase K; *L. johnsonii* La1 supernatant + Pronase: treatment with pronase; *L. johnsonii* La1 supernatant + Catalase: treatment with catalase) or heated at 90°C for 10 min before *G. duodenalis* growth inhibition assay. Growth inhibition (%) was normalized according to matched control: lactic acid-adjusted MTYI medium incubated with protease-coupled beads or treated for 10 min at 90°C. **(B)** Supernatant from *L. johnsonii* La1 in MTYI medium was adjusted to pH 6.2, 6.7, 6.9, or 7.2 before *Giardia* growth inhibition assays. Growth inhibition (%) was normalized according to control, i.e., lactic acid-adjusted MTYI subsequently raised to pH 6.2, 6.7, 6.9, or 7.2. Values are the mean ± SD of three independent experiments. **(C)** Molecular weight determination of inhibitory compounds from *L. johnsonii* La1 supernatant. Supernatant from a culture of *L. johnsonii* La1 in KM-FCS was filtrated through 10, 30, or 50 kDa MW cut-off membranes. Acidified KM-FCS alone was processed similarly. Fractions above and under respective thresholds were assayed for *Giardia* growth inhibition in the presence of bile (0.5 g/L). Inhibition values (%) were normalized according to KM-FCS controls. Data are the mean ± SD of three independent experiments performed in triplicate. Letters indicate significant differences between treatments (Kruskal-Wallis, *p* < 0.05).

To biochemically characterize the inhibitory activity present in *L. johnsonii* La1 supernatant, the supernatant was treated with immobilized enzymes prior to contact with parasites. Trophozoite growth inhibition was totally abolished by proteinase K and pronase treatments, suggesting involvement of inhibitory factor(s) of peptidic nature (Figure 2A). Heat-treatment also led to inactivation of *L. johnsonii* La1 supernatant inhibitory properties (Figure 2A). Additionally, in a pH range similar to the ones experienced by *G. duodenalis in vivo* (Biagini et al., 2001), a strong pH influence on the inhibitory activity was noticed, with the highest inhibition occurring at pH 6.2 (Figure 2B).

Moreover, Pridmore et al. (2008) demonstrated *L. johnsonii* La1 supernatant anti-*Salmonella* activity was mediated by the toxic effects of hydrogen peroxide (H_2_O_2_) and can be abolished by pretreatment with catalase. Therefore, we checked whether treatment of *L. johnsonii* La1 supernatant with catalase might prevent its anti-*Giardia* activity. Catalase pretreatment only slightly affected the inhibitory activity of *L. johnsonii* La1 supernatant on *G. duodenalis* (Figure 2A), invalidating the role of H_2_O_2_ in *Giardia* growth inhibition.

To assess whether *G. duodenalis* growth inhibition by *L. johnsonii* La1 supernatant might be due to toxic free fatty acids as demonstrated for *G. duodenalis* killing by human milk (Rohrer et al., 1986), FCS, bile, and *L. johnsonii* La1 supernatant were analyzed for NEFA content either alone or in combination, using the NEFA-C methodology (WAKO diagnostics). Then different samples were incubated for 24 h at 37°C with or without *G. duodenalis* trophozoites and cell supernatants were analyzed for NEFAs. Survival and growth of the parasites in those different conditions were determined.

KM medium with FCS or *L. johnsonii* La1 supernatant, displayed NEFA concentrations of 37.7 and 39.5 μM (oleic acid equivalents), respectively (Table 1). Co-incubation of bile with serum led to increased NEFA concentration (101.2 μM) suggesting that the increase might result from improved exposure of protein-bound fatty acids from serum by the action of bile acids, thus facilitating substrate (fatty acid) recognition by enzymes from the NEFA kit. The highest concentration of NEFAs (134.6 μM) was measured upon co-incubation of FCS with bile and *L. johnsonii* LA1 supernatant, as expected from summing their respective NEFA contents. It was verified that the presence of *G. duodenalis* had no noticeable effect on the NEFA content of the various samples (Table 1).

**Table 1:** **Involvement of non-esterified fatty acids in the *G. duodenalis* inhibition by *L. johnsonii* La1 supernatant**.

**Incubation medium**	**NEFAs (μM Eq) in presence of *Giardia***	**NEFAs (μM Eq) in the absence of *Giardia***	**Survival rate (%) in the presence of *Giardia***
KM	0	1.5 ± 1.4	21.4 ± 15.7
KM + FCS	37.7 ± 4.1	25.4 ± 8.3	94.7 ± 4.3
KM + *L. johnsonii* La1 supernatant	39.5 ± 6.8	37.4 ± 17.3	50.0 ± 20.7
KM + FCS + bile	101.2 ± 6.8	87.7 ± 5.1	97.1 ± 2.7
KM + FCS + *L. johnsonii* La1 supernatant	66.4 ± 4.2	60.1 ± 15.7	91.7 ± 3.7
KM + FCS + bile + *L. johnsonii* La1 supernatant	134.6 ± 5.5	149.5 ± 16.8	0

*The various components of the Giardia culture medium and L. johnsonii La1 supernatant were analyzed either alone or in combination for NEFA content after 24 h of incubation at 37°C. Incubation was performed with or without Giardia trophozoites (4.8 × 10^4^ / ml). At the end of the incubation period, survival and multiplication of the trophozoites in the different conditions were determined. Trophozoite survival rate after 24 h of incubation was expressed as the (number of living trophozoites / total number of trophozoites) × 100. Values are the mean ± SD from three independent experiments. It was verified that NEFA contents after 24 h of incubation at 37°C were similarly independent of the presence of Giardia*.

From the results in Table 1, it can be noted that normal parasite growth and survival (97.1%) observed in KM-FCS-bile was totally inhibited by addition of *L. johnsonii* La1 supernatant which induced 100% of parasite death, although the NEFA concentration was increased by only 1.33X (i.e., from 101.2 to 134.6 μM). This strongly suggested that NEFAs do not play a prominent role in *Giardia* inhibition by *L. johnsonii* La1 supernatant.

#### *L. johnsonii* extracellular products inhibiting *Giardia* growth are>10 kDa factor(s)

Fractionation experiments of *L. johnsonii* La1 supernatant by ultrafiltration using different molecular weight cut-offs indicated that the *L. johnsonii* La1 supernatant inhibitory activity was due to molecule(s) bigger than 10 kDa, since the fraction >10 kDa showed a much higher *G. duodenalis* inhibition compared to the fraction < 10 kDa (around 70 vs. 20% inhibition, respectively, *p* < 0.05, Figure 2C). By performing a 30 kDa threshold fractionation, the inhibitory activity concentrated mostly in the >30 kDa fraction (66% of *Giardia* growth inhibition, even if not significantly different from the control, *p* > 0.05, Figure 2C), however an inhibitory effect (~40%) was also observed with the < 30 kDa fraction. A 50 kDa threshold fractionation of *L. johnsonii* La1 supernatant was unable to segregate the inhibitory activity, i.e., 50 and 48% of *Giardia* growth inhibition were induced by < 50 and >50 kDa fractions, respectively (Figure 2C).

Fractionation of *L. johnsonii* La1 supernatant was also realized by dialysis using a 3.5-kDa cut-off membrane against KM-FCS or GKN buffer (Pérez et al., 2001). Parasite killing activity of *L. johnsonii* La1 supernatant in the presence of bile was fully recovered after dialysis, whatever the dialysis solution, indicating that no element crucial to the inhibitory activity was lost upon dialysis (Table 2). All together, these data indicated that *L. johnsonii* La1 supernatant factor(s) involved in the inhibitory activity display molecular weight > to 10 kDa.

**Table 2:** **Dialysis through 3.5 kDa cut-off membrane does not inactivate the inhibitory activity of *L. johnsonii* La1 supernatant**.

	**KM-FCS**	***L. johnsonii* La1 supernatant**	***L. johnsonii* La1 supernatant dialyzed against KM-FCS**	***L. johnsonii* La1 supernatant dialyse against GKN**
**GROWTH INHIBITION ASSAY IN THE ABSENCE OF BOVINE BILE**				
Multiplication factor	3.8	3.2	3.7	2.4
Survival rate (%)	93.7	88.3	90.2	89.3
**GROWTH INHIBITION ASSAY IN THE PRESENCE OF BOVINE BILE**				
Multiplication factor	2.3	1.2	1.5	0.8
Survival rate (%)	92.9	0	0	0

*L. johnsonii La1 supernatant was dialyzed against GKN buffer or KM-FCS and then tested for G. duodenalis trophozoite growth in the presence or absence of bovine bile (0.5 g/L). Non-dialyzed KM-FCS and L. johnsonii La1 supernatant were used as controls. Parasite multiplication factor was expressed as the number of total trophozoites / number of trophozoites at time zero of incubation. Trophozoite survival rate was expressed as the (number of living trophozoites / total number of trophozoites) × 100. Values are the mean of two independent experiments performed in duplicate*.

### *L. johnsonii* La1 supernatant modifies bile composition by increasing deconjugated bile salts that are toxic to *Giardia*

**Figure 3:**
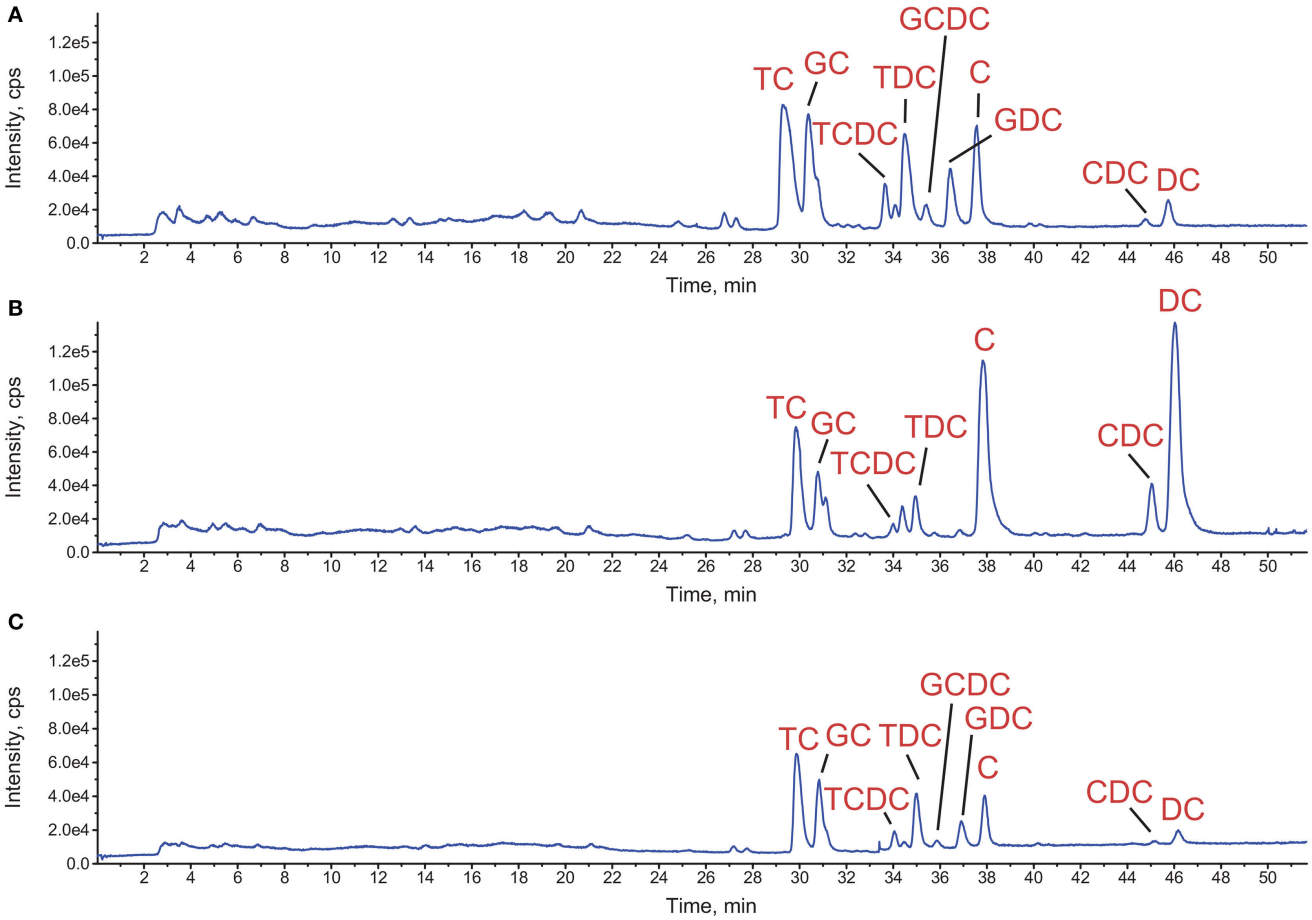
**Impact of *L. johnsonii* La1 supernatant on bovine bile composition**. Total ion chromatograms detected by LC-MS of the treated samples corresponding to incubation of bile for 24 h at 37°C with either **(A)** KM alone, **(B)** KM + *L. johnsonii* La1 supernatant or, **(C)** KM + heat-treated *L. johnsonii* La1 supernatant (10 min, 90°C). TC, taurocholate; GC, glycocholate; TCDC, taurochenodeoxycholate; TDC, taurodeoxycholate; GCDC, glycochenodeoxycholate; GDC, glycodeoxycholate; C, cholate; CDC, chenodeoxycholate; and DC, deoxycholate.

**Figure 4:**
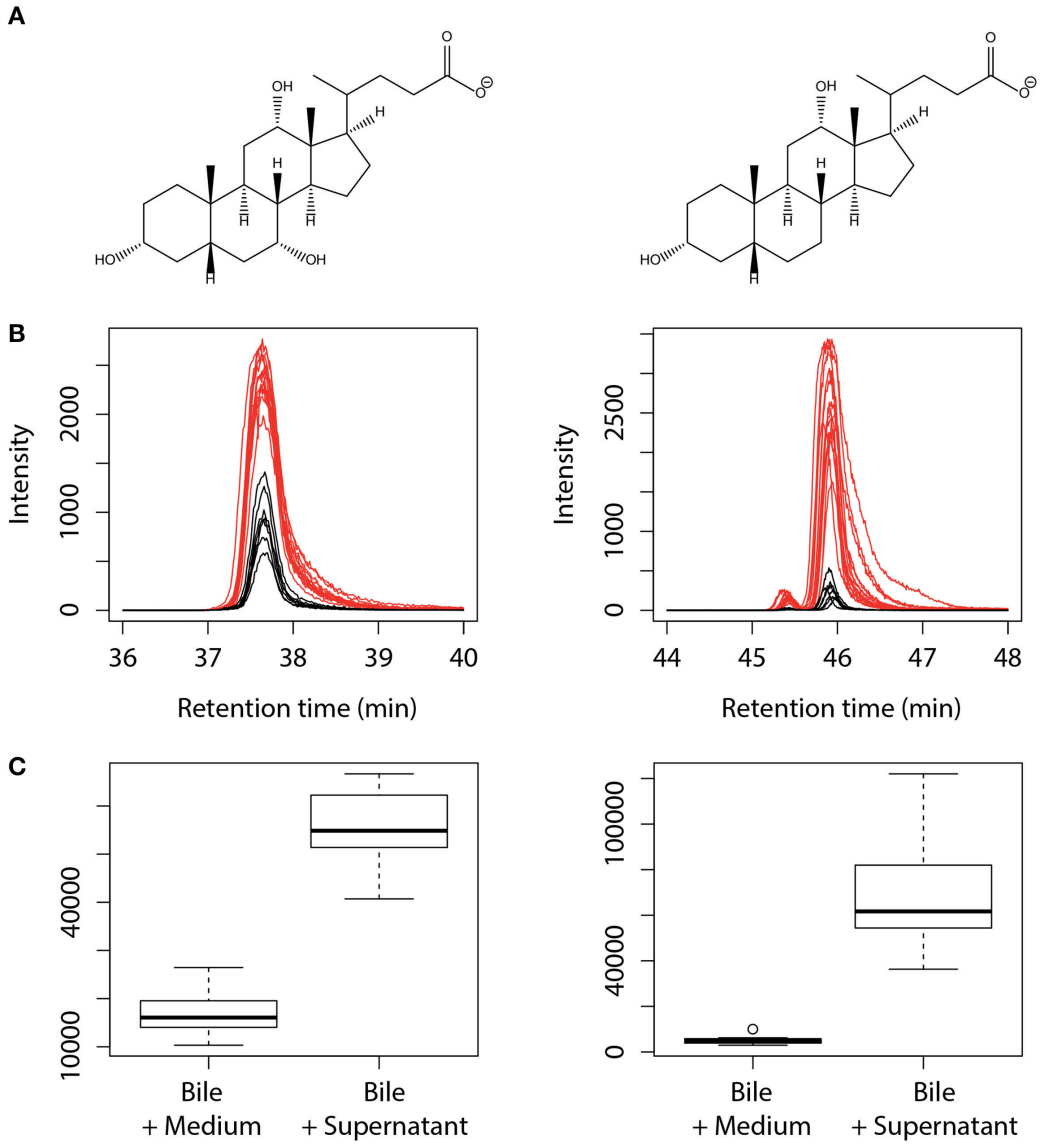
**Increase of the non-conjugated salts cholate and deoxycholate content after treatment of bovine bile with *L. johnsonii* La1 supernatant**. **(A)** Structures of cholate ([M-H]- at *m/z* 407, left side) and deoxycholate ([M-H]- at *m/z* 391, right side). **(B,C)** Corresponding extracted ion chromatograms **(B)** and peak area boxplot **(C)** obtained from bile samples incubated with KM (in black) or *L. johnsonii* La1 supernatant (in red).

Since concomitant addition of bovine bile and *L. johnsonii* La1 supernatant to the culture medium leads to inhibition of *G. duodenalis* growth, we assessed whether bile composition might be modified by *L. johnsonii* La1 supernatant. Bile composition was investigated by LC/ESI-MS after 24 h of incubation with *L. johnsonii* La1 supernatant (Figures 3A,B). Impacted molecules were identified by their *m/z* MS/MS fragmentation pattern and comparison with standards. Comparison of bile salt profiles showed a decrease of conjugated salts (GC, TC, GDC, TDC, GCDC, TCDC) in favor of non-conjugated salts. C and DC were the main statistically enhanced non-conjugated salts (Figure 4) and in a minor proportion, CDC. These modifications were not observed in presence of heat-treated *L. johnsonii* La1 supernatant (Figure 3C).

The inhibition of *G. duodenalis* growth by pure bile salts (C, DC, and CDC) conjugated to glycine or taurine, or their deconjugated counterparts were investigated in the presence or the absence of *L. johnsonii* La1 supernatant (Table 3). In the absence of *L. johnsonii* La1 supernatant, glycyl-, or tauryl-conjugated salts and deconjugated C showed no apparent toxicity at the concentrations tested (IC_50_ values >400 μM). In contrast, deconjugated DC, and CDC exhibited inhibitory effects on trophozoite growth with IC_50_ values of 132 μM (DC) and 147 μM (CDC). Interestingly, in the presence of *L. johnsonii* La1 supernatant, the conjugated bile salts IC_50_ values fell to a range of values similar to those measured for their pure deconjugated counterparts, i.e., 104 (GDC), 79 (TDC), 110, (GCDC), and 115 μM (TCDC) (Table 3).

**Table 3:** **Conjugated bile salts in association with *L. johnsonii* La1 supernatant as well as deconjugated bile salts prevent the growth of *G. duodenalis***.

		**KM + FCS IC_50_ (μM)**	**KM + FCS + *L. johnsonii* La1 supernatant IC_50_ (μM)**
C	Cholate	>400	>400
GC	Glycocholate	>400	>400
TC	Taurocholate	>400	>400
DC	Deoxycholate	132 ± 13	117 ± 13
GDC	Glycodeoxycholate	>400	104 ± 13
TDC	Taurodeoxycholate	>400	79 ± 17
CDC	Chenodeoxycholate	147 ± 15	118 ± 22
GCDC	Glycochenodeoxycholate	>400	110 ± 11
TCDC	Taurochenodeoxycholate	>400	115 ± 8
FA	Fusidic acid	26 ± 4	nd.

*IC_50_ estimations. Various concentrations of taurine—and glycine-conjugated and unconjugated C, DC and CDC bile salts were tested for G. duodenalis trophozoite growth inhibition in KM-FCS in the presence or absence of L. johnsonii La1 supernatant. IC_50_ values were determined from drug-response curves and expressed as mean ± SD of at least three independent experiments. nd.: not determined*.

These results suggested that a deconjugating process mediated by *L. johnsonii* La1 supernatant component(s) and production of deconjugated bile salts might be responsible for the inhibitory effect of the association of bile with *L. johnsonii* La1 supernatant. Such a hypothesis is in line with the previous observation that fusidic acid, an antibiotic with a bile salt-like chemical structure, is toxic to *Giardia* (see Table 3, IC_50_value = 26 μM) unless conjugated to taurine or glycine (Farthing and Inge, 1986).

### Potential involvement of *L. johnsonii* La1 bile-salt hydrolase(s) activity in bile-mediated *Giardia* inhibition

#### Correlation between bile salt hydrolase activity and anti-giardial properties after fractionation of extracellular products

It is known that the bile salt deconjugating process is mediated by 3-alpha, 7-alpha, 12-alpha-trihydroxy-5-beta-cholan-24-oylglycine / taurine amidohydrolases (EC 3.5.1.24), also named choloylglycine / taurine hydrolases, conjugated bile acid hydrolases (CBAH), or BSH (Nair et al., 1967). These enzymes act on non-peptide carbon-nitrogen bonds, specifically in linear amides, releasing glycine and taurine from conjugated bile salts. Three genes (LJ0056, LJ1147, and LJ1412) encoding BSH-like enzymes have been predicted in the genome of *L. johnsonii* (Morrison et al., 2007). These enzymes are also predicted to be secreted, as indicated using SecretomeP (predictions of a non-classical i.e., not signal peptide triggered protein secretion), with theoretical full sizes of 34.9, 36.3, and 36.6 kDa and secreted sizes of 29.8, 31.0, and 31.3 kDa, respectively.

**Figure 5:**
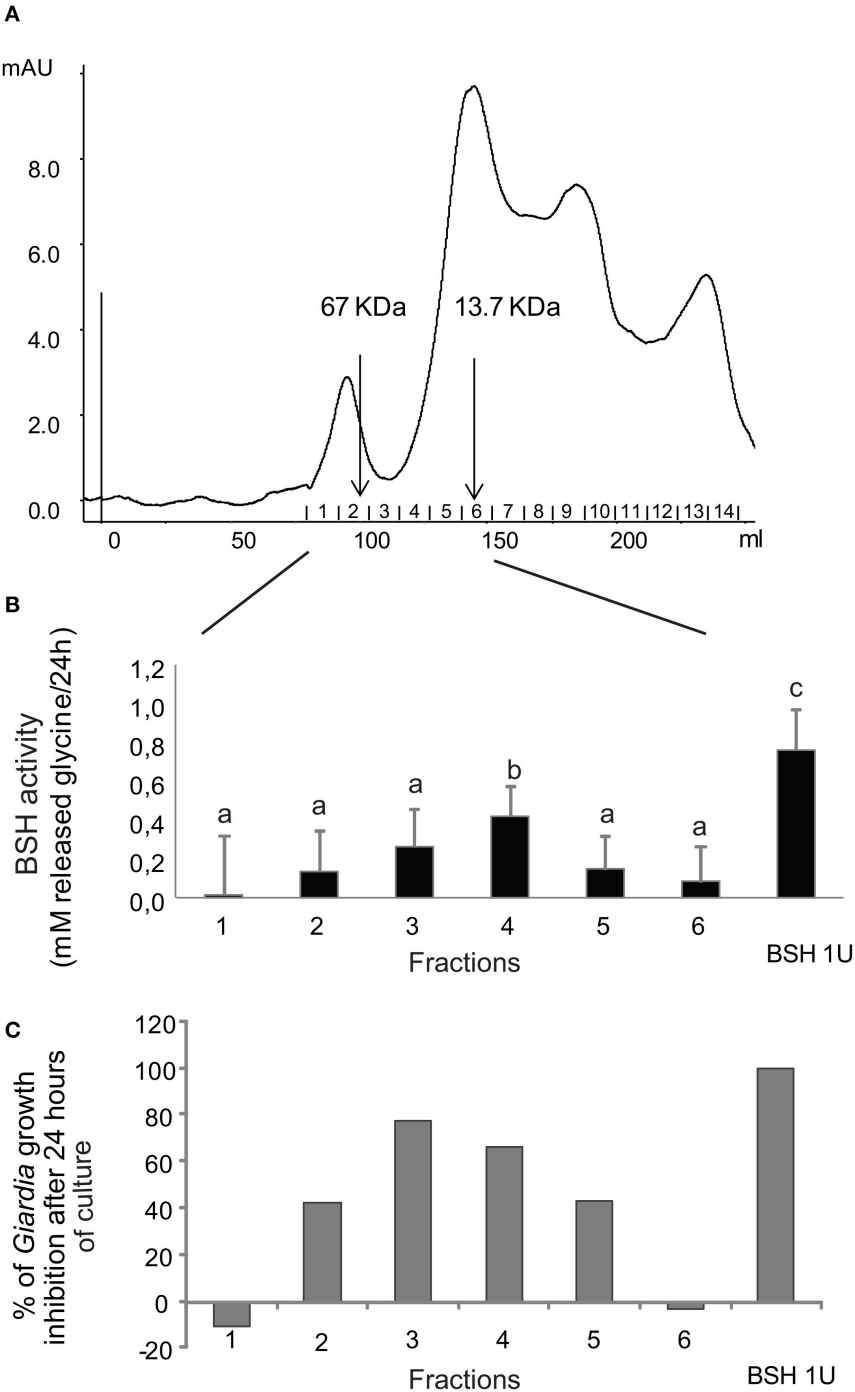
**Inhibitory activity against *G. duodenalis* growth and BSH-like activity co-elute in the same fractions after separation of *L. johnsonii* La1 supernatant by gel filtration chromatography**. **(A)** Chromatography profile. **(B)** BSH-like activity measured after 24 h of incubation of GDC with the 6 first eluated gel filtration fractions. **(C)** *G*. *duodenalis* growth inhibitory activity after 24 h of incubation with the same gel filtration chromatography fractions as in B. Letters indicate significant differences between treatments (Kruskal-Wallis, *p* < 0.05).

To test the hypothesis of bacterial BSH involvement in the inhibitory activity of *L. johnsonii* La1 supernatant, the bacterial supernatant was fractionated by gel filtration chromatography on a Sephacryl S300 column, and eluted fractions were assayed for both parasite inhibition and bile salt deconjugating activity. A typical elution profile is shown in Figure 5A. Fractions were collected, tested for *G. duodenalis* growth inhibition and for BSH-like activity by measuring release of glycine from GDC. In our experimental conditions, 1 unit of the commercial *C. perfringens* BSH induced the release of 0.76 mM glycine from 2.5 mM GDC within 24 h. Parasite growth inhibitory activity was reproducibly recovered from the first eluted fractions containing proteins with molecular weight between 13.7 and 67 kDa (Figures 5A,C) confirming the estimate of the molecular weight of the inhibitory factor(s) to be >10 kDa (Figure 2C). Those active fractions also exhibited a BSH-like activity (Figure 5B), with the most inhibitory fractions showing significantly higher BSH-like activity (*p* < 0.05). Our attempts to further purify elements responsible for the inhibitory activity and/or BSH-like activity by combining steps of ion-exchange, hydrophobic interaction and chromatofocusing chromatographies were unsuccessful, with a rapid loss of activities.

#### Active *Clostridium perfringens* BSH in presence of tauryl and glycyl-bile salts mimics the anti-giardial activity of *L. johnsonii* extracellular fractions

**Figure 6:**
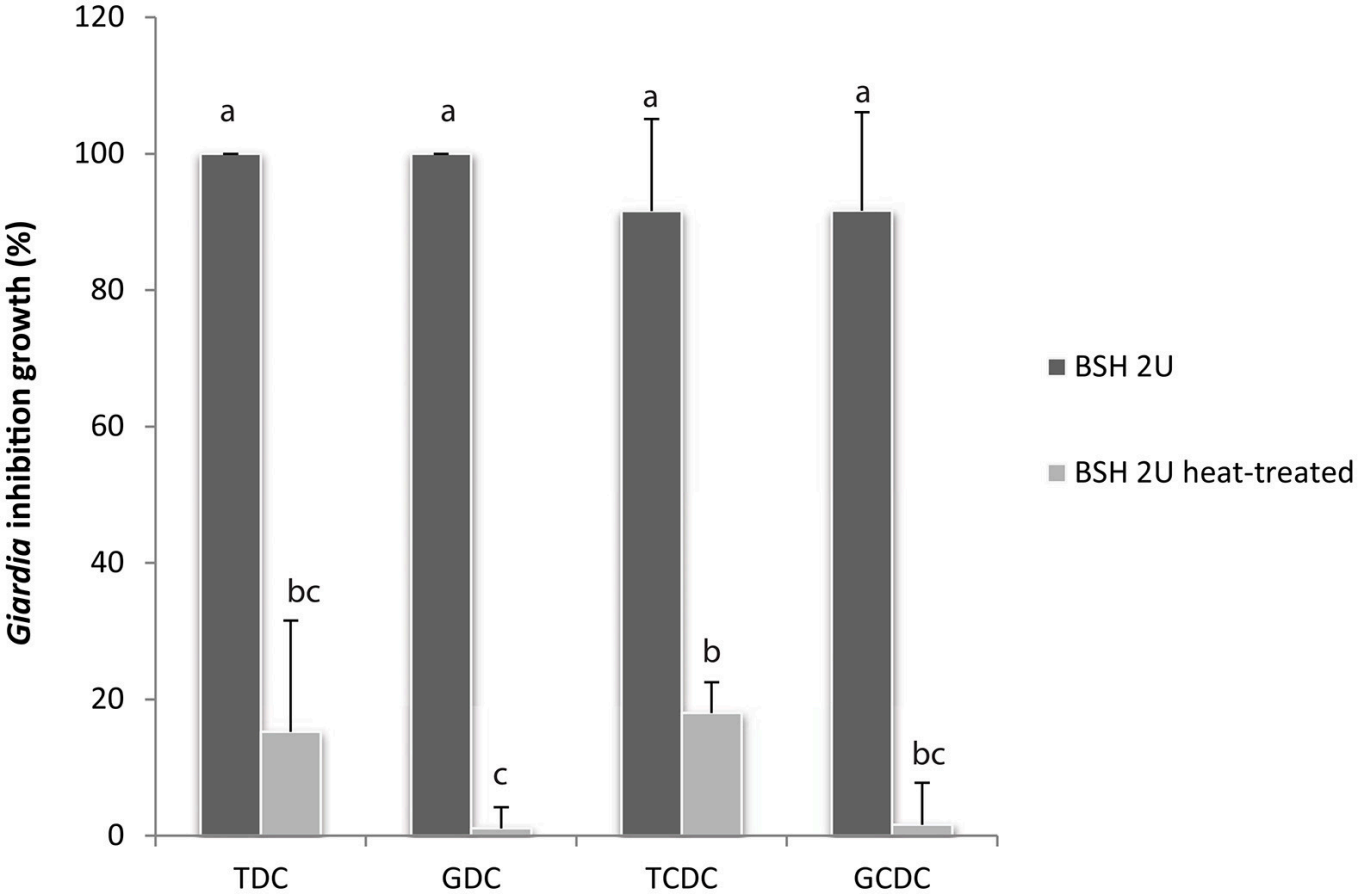
***G*. *duodenalis* growth is inhibited by enzymatically active *C. perfringens* BSH in the presence of conjugated bile salts (0.2 g/L)**. Commercial *C. perfringens* BSH, enzymatically active or heat-inactivated (100°C, 5 min) was added to *G. duodenalis* culture medium in the presence of either GDC, glycodeoxycholate; GCDC, glycochenodeoxycholate; TDC, taurodeoxycholate or TCDC; taurochenodeoxycholate. Values are the mean ± SD of at least three independent experiments. Letters indicate significant differences between treatments (Kruskall-Wallis, *p* < 0.05).

To assess the capability of BSH enzymes to promote the bile-mediated anti-*Giardia* effect, BSH from the bacteria *C. perfringens* was tested for *G. duodenalis* growth inhibition in the presence of bile or pure conjugated bile salts. As notified (Table 3), glycine or taurine conjugated bile salts, TDC, TCDC, GDC, and GCDC have no inhibitory activity on *G. duodenalis* growth in KM-FCS. In contrast, the addition of *C. perfringens* BSH to the culture in the presence of the conjugated bile salts led to a remarkable parasite inhibition within the 24 h of the assay, with inhibition ranges of 95–100% depending on the conjugated bile salt (Figures 5B, 6). Heat inactivation of *C. perfringens* BSH (100°C, 5 min) dramatically reduced or even abolished its anti-*Giardia* activity (less than 20% growth inhibition depending on the conjugated bile salts tested, Figure 6) indicating that parasite growth inhibition depends on BSH enzymatic activity.

#### Evidence for the presence of at least two BSH-like enzymes in the *L. johnsonii* La1 supernatant

We investigated whether the BSH-like enzymes annotated from *L. johnsonii* La1 genome and predicted to be secreted are indeed released into the bacterium culture medium. High-resolution mass spectrometry-based proteomic analysis of *L. johnsonii* La1 extracellular proteins was performed. Three micro gram of proteins from two independent culture supernatants allowed high-confidence identification of 1787 and 1746 peptides (FDR < 1% at peptide level and only rank 1 peptide identification) providing a total of 164 and 166 protein groups with a minimum of 2 high-confidence peptides per protein, respectively (Table S1) and 147 common proteins between the two replicates (Figure S2). Amongst them, two of the three predicted BSHs, LJ1412 (gi|41583570), and LJ1147 (gi|41583360), were clearly identified by an untargeted/undirected approach in both replicates (Table 4).

**Table 4:** **Identification of bile salt hydrolases in *L. johnsonii* La1 culture supernatant by MS/MS peptide fragmentation using MASCOT stringent search**.

**Accession number^a^**	**Score^b^**	**Cov. (%)^c^**	**Unique peptides**	**Total peptides**	**PSMs**	**Peptide ion sequence^d^**	**Charge^e^**	**Peptide ion (m/z)**	**Theo. Mass (kDa)^f^**	**Theo. p*I*^g^**
**EXPERIMENTAL REPLICATE 1**										
gi|41583570	162	12.58	2	2	5	NLANYSNIAPAQPK AHSPQGNNELSSVTNYFHILHSVEQPK	2 4	750.9 759.1	36.6	5.2
gi|41583360	39	12.92	3	3	3	GLGIAGLNFTGPGK DLPVTTLHWLMGDK NTLVPNADINLYSR	2 2 2	651.4 813.4 795.4	36.3	4.9
**EXPERIMENTAL REPLICATE 2**										
gi|41583570	83	12.58	2	2	3	NLANYSNIAPAQPK AHSPQGNNELSSVTNYFHILHSVEQPK	2 4	750.9 759.1	36.6	5.2
gi|41583360	77	8.62	2	2	3	GLGIAGLNFTGPGK NTLVPNADINLYSR	2 2	651.4 795.4	36.3	4.9

a Accession number in the NCBI protein database. All accession numbers refer to sequences from Lactobacillus johnsonii NCC 533;

b Probability-based Mowse score of MASCOT software that evaluates if the peptides subjected to search are the same as those found in the database originated by in silico digestion of a known protein;

c Coverage (Cov.) is the percentage of predicted protein sequence covered by matched peptides via MASCOT;

d Peptide sequences identified via MASCOT following the experimental peptide masses after parental ion fragmentation;

e Doubly- to quintuply-charged ions of selected peptides were detected;

f Theoretical molecular masses of proteins calculated from amino acid sequences;

g *Theoretical isoelectric points of proteins calculated from amino acid sequences*.

## Discussion

Probiotics constitute promising protective alternatives against pathogens, at least as prophylactic agents in various diseases or when used in combination with other therapeutic agents, through a variety of mechanisms relying on immunomodulation properties, competition for nutrients, and habitats, and secretion of active molecules (e.g., antimicrobial peptides, bacteriocins, antibiotics, free fatty acids, hydrogen peroxide; For reviews, Britton and Versalovic, 2008; Travers et al., 2011). In the context of *Giardia* infections, a number of studies pointed out the importance of the microbiota and the potential role of probiotics in preventing infection and development of disease (Singer and Nash, 2000; Humen et al., 2005; Shukla et al., 2008, 2012; Shukla and Sidhu, 2011; Goyal et al., 2013; Goyal and Shukla, 2013). *L. johnsonii* La1 (NCC533), *Lactobacillus casei* MTCC1423, and *E. faecium* SF68 were consistently identified throughout these studies. In *in vivo* experiments, those strains were shown to prevent parasite-induced intestinal damage and decrease duration and severity of giardiasis (Humen et al., 2005; Shukla et al., 2008, 2012; Shukla and Sidhu, 2011). Moreover, Pérez et al. (2001) reported inhibitory activity of *L. johnsonii* La1 on *in vitro* growth of *G. duodenalis* trophozoites. A partial characterization of the factors involved in the anti-giardial action indicated heat-inactivable low-molecular weight (< 1 kDa) components.

Based on this evidence and the potential of probiotics to regulate *Giardia* proliferation, we aimed to decipher the molecular mechanisms involved in the *in vitro* anti-giardial activity of *L. johnsonii* La1. In an experimental assay similar to that published by Pérez et al. (2001), we were able to confirm that *L. johnsonii* La1 supernatants significantly inhibit the proliferation of different strains of *G. duodenalis in vitro*. Pérez et al. (2001) showed that the inhibitory effect of *L. johnsonii* La1 supernatant upon *G. duodenalis* growth was pH dependent with a more pronounced inhibitory effect at pH 6 than pH 7. Inhibitory assays were thus performed at pH 6 instead of pH 6.8–7, the pH normally used for *Giardia* culture. The pH change had a slight effect on parasite proliferation and adhesion properties (Pérez et al., 2001). We also observed no effect on the parasite morphology, motility and viability (Video S1, Figure S1). Furthermore, to avoid an inhibitory activity due to the acidification with lactic acid produced by *L. johnsonii La1*, bacteria supernatants were neutralized before the assays and appropriate controls were prepared by adding equivalent of lactic acid to fresh medium before pH adjustment.

Using biochemical fractionations and assays, we were able to document the properties of an active component present in the *L. johnsonii* La1 supernatant that is inhibitory toward *Giardia* trophozoites. We first showed that this active(s) component(s) is lactic acid-independent, does not rely on free fatty acids, and is distinct from H_2_O_2_. By using dialysis, ultrafiltrations and combinations of enzymes, we showed that this active(s) component(s) is of peptidic nature, thermosensitive and has a molecular weight >10 kDa. We also demonstrated correlations between this active(s) component(s) inhibiting *Giardia* and (1) production of deconjugated bile salts toxic to *Giardia* and (2) BSH activity(ies) of bacterial origin. However, since lactobacilli are known to secrete a large variety of molecules (Lebeer et al., 2008; Cicenia et al., 2014) that could act in concert to bring this antimicrobial effect, and since the active component evidenced by Perez was < 1 kDa, we cannot exclude the existence of other factors, secreted or released by *L. johnsonii* La1, that might also contribute to *Giardia* inhibition.

We bring here a body of evidence strongly suggesting BSH-like activities in the anti-giardial effect: (1) we observed a conversion of conjugated bile salts into their toxic deconjugated counterparts by *L. johnsonii* La1-supernatant, (2) a similar effect was mimicked by purified *Clostridium* BSH, (3) we identified two BSHs in *L. johnsonii* La1 supernatant by mass spectrometry and (4) we measured BSH-like activities in the *L. johnsonii* La1 fractionated supernatant, with the most enzymatically active fractions mediating the highest toxicity to *Giardia*.

BSHs (EC 3.5.1.24) are bacterial enzymes known to hydrolyze the amide bond in the C24 position of a wide variety of conjugated bile salts, releasing a deconjugated salt and a taurine or glycine residue (For review, Begley et al., 2006). BSHs are mainly expressed by Gram+ bacteria such as *Lactobacillus, Bifodobacterium* and *Enterococcus* species, although some species do not express them. BSH activity allows for the detoxification of conjugated bile salts, which are amphipathic molecules with surfactant properties that can be toxic toward bacterial cells (De Boever and Verstraete, 1999). It is believed that bacterial BSH production is involved in the maintenance of their environment, notably in the small intestine (for the probiotic strains) where bile concentration is at its highest (Begley et al., 2006). This is the first proposal for bacterial BSH activity regulating parasite survival. Formal identification of the specific BSH(s) implicated in this phenomenon is required. This may be accomplished by recombinant protein production and/or invalidation of the *L. johnsonii* La1 BSH genes (Pridmore et al., 2004). As previous enzymatic studies on BSHs revealed that they may have distinct affinities and specificities for glyco- or tauro- conjugates (For review, Ridlon et al., 2006), it is important to define whether anti-*Giardia* activity is mediated by a single type of BSH with a defined specificity, or by several BSH enzymes with mixed specificities. BSHs from human intestinal lactobacilli usually have the highest affinity for glycine conjugates (Ridlon et al., 2006).

We demonstrate here a two-step mechanism in which a probiotic transforms bile components that could be toxic for itself into components that are toxic to *Giardia* trophozoites. *Giardia* tolerance to conjugated bile salts *in vitro* for concentrations that are toxic to mammalian cell cultures relies on transport-mediated processes (Halliday et al., 1995). At least two distinct uptake mechanisms have been described, one Na^+^-dependent and thiol blocker sensitive for cholyltaurine and the other one Na^+^- and thiol blocker insensitive for cholylglycine. Both uptake mechanisms are counterbalanced by a rapid efflux mechanism regulating the intracellular concentration of these detergents to below their toxicity to *Giardia* (Das et al., 1997). No biotransformation of conjugated bile salts into their deconjugated counterparts by *Giardia* was observed. The authors suggested that intracellular bile salts may facilitate lipid trafficking and membrane biosynthesis in *Giardia*. Indeed, *Giardia* lipid-binding proteins have been shown to have higher affinity for bile salts than fatty acids, suggesting that lipids are incorporated by these binding proteins more readily inside the micelles formed by bile salts than by free fatty acids (de la Guardia et al., 2011). Studies also suggest that conjugated bile salts have a role in promoting *Giardia* encystation (Gillin, 1987; Gillin et al., 1989), a vital step in the parasite life cycle that enables parasite survival outside of its host. It could be of interest to find out whether such mechanisms can also act on the parasite development cycle (encystation and cyst properties). In this study, experiments were performed using bovine bile instead of using human bile as both species have similar proportions of cholic, desoxycholic, and chenodesoxycholic acids. Besides, human, and bovine bile exhibit an equivalent ratio of glycoconjugated bile salts over their tauroconjugated counterparts (3:1) (Zhong et al., 1998). It can thus be expected a similar inhibitory activity with human bile.

Several studies mentioned briefly the toxic effect of deconjugated bile salts on *Giardia* (Farthing et al., 1983, 1985, 1987; Gillin, 1987). To our knowledge, our study is the first to demonstrate their dose-dependent anti-giardial activity. Their IC_50_ values are lower than their critical micellar concentrations, indicating that parasite killing is not related to their surfactant properties (e.g., IC_50_ value for chenodeoxycholate = 0.15 mM and a critical micellar concentration >7 mM according to the manufacturer). Their mechanism of action remains to be deciphered. Interestingly, their inhibitory activity seems dependent of their hydrophobic properties. No inhibition was observed with the most hydrophilic salt, cholate (IC_50_ value >400 μM), unlike the more hydrophobic salts, deoxycholate and chenodeoxycholate (IC_50_ values of 132 and 147 μM, respectively). Our results are in line with the previous observation that the bile salt-like antibiotic, fusidic acid, is toxic to *Giardia* (Farthing and Inge, 1986), which we confirmed (IC_50_ = 26 μM). However, its taurine and glycine conjugates stimulate parasite growth at low concentrations, while modest growth inhibition occurred at higher concentrations.

In conclusion, we demonstrate that the inhibitory effect exerted *in vitro* by *L. johnsonii* La1 supernatant on *Giardia* growth (1) is dependent on the presence of bile in the growth medium, more precisely bile salts, (2) relies on molecules released by *L. johnsonii* La1 that are of peptidic nature with a molecular weight >10 kDa, (3) is due to the conversion of conjugated bile salts into deconjugated bile salts that are toxic to *Giardia*, (4) correlates with the presence and activity of BSH-like enzymes from bacterial origin and (5) can be reproduced by addition of *C. perfringens* BSH to the culture medium. These experimental results point out that BSHs released by *L. johnsonii* La1 would be main facilitators of this anti-parasitic effect. These discoveries open a novel field to fight *Giardia* based on the use of deconjugated bile salts, chemically related drugs or by administration of BSH-releasing probiotic strains.

## Author contribution

Conceived and designed the experiments: MT, CS, IF, and PG. Performed the experiments: MT, CS, SZ, CD, SoC, RQ, SeC, IF, and PG. Analyzed the data: MT, CS, SZ, CD, RQ, SeC, IF, and PG. Wrote the paper: MT, CS, TA, IF, and PG.

## Funding

This work was supported by Région Ile de France, Programme Interdisciplinaire CNRS, “Maladies Infectieuses Emergentes,” DIM Maladies Infectieuses, Parasitaires et Nosocomiales Émergentes (progamms n° 90212 and DIM 120092), Interdisciplinary Programs of the MNHN (ATM-Microorganismes), the CAPES-COFECUB program [723/11] and CNPq (Conselho Nacional de Desenvolvimento Cientiífico e Tecnológico).

### Conflict of interest statement

The authors declare that the research was conducted in the absence of any commercial or financial relationships that could be construed as a potential conflict of interest.

## Supplementary material

Video S1 **Trophozoites incubated 24 h in KM, pH 6, 0.6 g/L bile salts (AVI)**.

Video S2 **Trophozoites incubated 24 h in KM, pH 6.0, 0.6 g/L bile salts and *L. johnsonii* La1 supernatant (AVI)**.

Figure S1 **Analysis by flow cytometry of *G. duodenalis* trophozoite viability after staining with propidium iodide (Word)**.

Figure S2 ***G*. *duodenalis* (WB and HP1 strains) growth inhibition by *L. johnsonii* La1 supernatant in the presence bovine bile from two commercial origins in KM-FCS medium (Word)**.

Figure S3 **Venn diagram between the two replicates of the *L. johnsonii* La1 supernatant (Word)**.

Table S1 **Proteome of *L. johnsonii* La1 supernatant (2 biological replicates) by MS/MS peptide fragmentation using MASCOT stringent search (XLSX)**.

## Abbreviations

BSH: bile salt hydrolase
GC: glycocholate
GCDC: glycochenodeoxycholate
GDC: glycodeoxycholate
KM: TYI-S-33 Keiser' medium
KM-FCS: KM with fetal calf serum
MTYI: modified TYI-S-33
NEFA: non-esterified fatty acids
TC: taurocholate
TCDC: taurochenodeoxycholate
TDC: taurodeoxycholate.
