# Supplementary material for: Deconjugated Bile Salts Produced by Extracellular Bile-Salt Hydrolase-Like Activities from the Probiotic Lactobacillus johnsonii La1 Inhibit Giardia duodenalis In vitro Growth
Source: Front Microbiol. 2016 Sep 27;7:1453. doi: 10.3389/fmicb.2016.01453 (PMC5037171; doi:10.3389/fmicb.2016.01453)

**S1 Fig : Analysis by flow cytometry of *G. duodenalis* trophozoite viability after staining with propidium iodide.**

The inhibitory effect of *L. johnsonii* La1 supernatant on *G. duodenalis* growth was measured by flow cytometry. Trophozoite viability was evaluated using propidium iodide (PI) and was assessed in addition to Malassez cell chamber counting. PI was used as a viability marker allowing identification of dead cells as it is excluded from viable cells (1). Parasites (1,5.10^5^ trophozoites) were maintained for 20 hours in KM medium, pH 6, in the presence (C, D) or the absence (A, B) of *L. johnsonii* La1 supernatant and with (B, D) or without (A, C) bovine bile.

Parasite viability was assessed on culture aliquots chilled on ice for 10 min by counting and examining their morphology and motility. More than 95% of parasites were considered as alive showing a typical pear shape and flagella mobility in KM, KM plus bovine bile and KM plus *L. johnsonii* La1 supernatant. In contrast, less than 5% were estimated alive when parasites were incubated in KM containing both bovine bile and *L. johnsonii* La1 supernatant.

For flow cytometry analysis, parasites were chilled on ice for 10 min, centrifuged at 1900 rpm for 5 min and the cells resuspended in 100 µl PBS containing 5 µg/mL PI. After 10 min of incubation in the dark, parasites were washed twice in PBS and fixed overnight at 4°C in a 3.7% formaldehyde solution. Analyses were performed using a FACSVerse Cytometer (BD Biosciences). Cytometric acquisition settings were defined using unstained cell sample for autofluorescence issues. Cells were evaluated upon size (Forward Scatter; FSC-A) and PI signal intensity (PE-A). PI staining correlates the viability estimated on morphology and motility with less than 5% of dead parasites in KM (A), KM plus bovine bile (B) and KM plus *L. johnsonii* La1 supernatant (C) conditions, and more than 95% of dead parasites in KM containing both bovine bile and *L. johnsonii* La1 supernatant (D).

1: Barbosa J, Costa-de-Oliveira S, Rodrigues AG, Pina-Vaz C.Optimization of a flow cytometry protocol for detection and viability assessment of *Giardia lamblia*. Travel Med. Infect. Dis. 2008, 6(4):234-9.


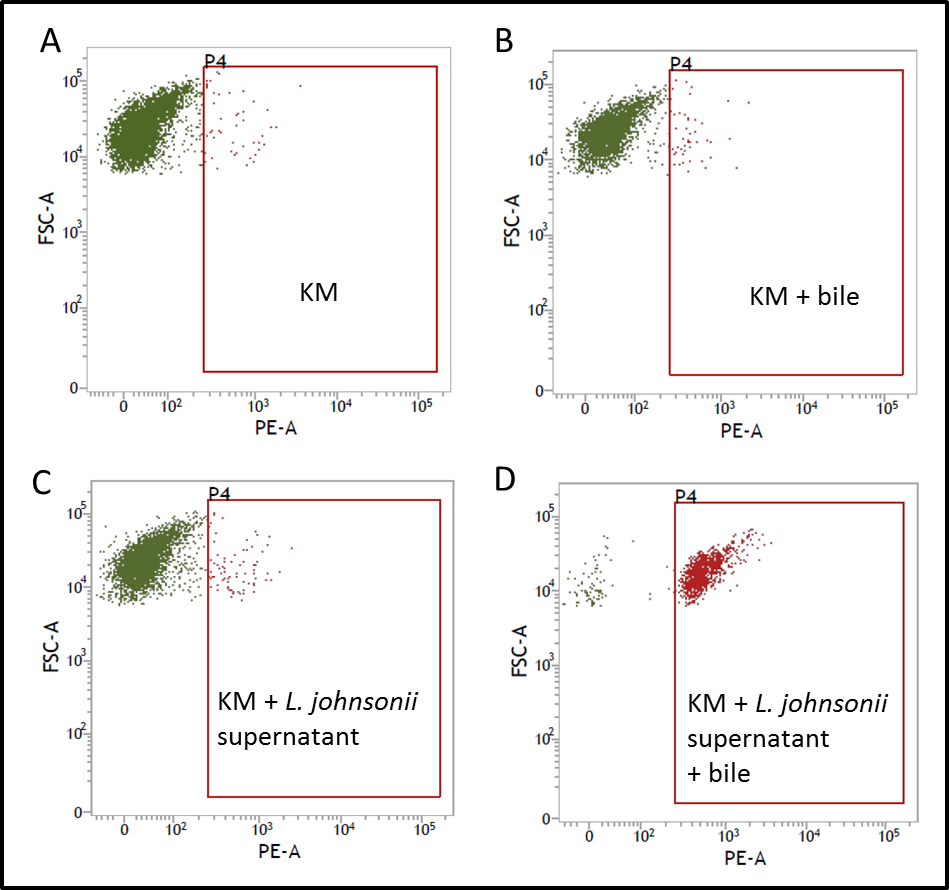

Supplement: Figure S1 — Analysis by flow cytometry of G. duodenalis trophozoite viability after staining with propidium iodide (Word). [file DataSheet1.docx]
