# Supplementary material for: Deconjugated Bile Salts Produced by Extracellular Bile-Salt Hydrolase-Like Activities from the Probiotic Lactobacillus johnsonii La1 Inhibit Giardia duodenalis In vitro Growth
Source: Front Microbiol. 2016 Sep 27;7:1453. doi: 10.3389/fmicb.2016.01453 (PMC5037171; doi:10.3389/fmicb.2016.01453)
Supplement: Figure S2 — G. duodenalis (WB and HP1 strains) growth inhibition by L. johnsonii La1 supernatant in the presence bovine bile from two commercial origins in KM-FCS medium (Word). [file DataSheet2.docx]

**S2 Fig. *Giardia duodenalis* (WB and HP1 strains) growth inhibition by *L. johnsonii* La1 supernatant in the presence bovine bile from two commercial origins, in KM-FCS medium.**

*G. duodenalis* trophozoites (strain WB and HP-1) were grown in KM-FCS with or without bovine bile (0.6 g/L, final concentration) in the presence or the absence of bacterial supernatant. The parasite concentration was estimated by counting live cells with a Malassez cell chamber. Growth inhibition values were normalized according to controls in lactic acid-acidified KM-FCS supplemented with similar concentrations of bovine bile. Values are the mean +/- SD of two independent experiments performed in triplicate.
