# Supplementary material for: Deconjugated Bile Salts Produced by Extracellular Bile-Salt Hydrolase-Like Activities from the Probiotic Lactobacillus johnsonii La1 Inhibit Giardia duodenalis In vitro Growth
Source: Front Microbiol. 2016 Sep 27;7:1453. doi: 10.3389/fmicb.2016.01453 (PMC5037171; doi:10.3389/fmicb.2016.01453)
Supplement: Figure S3 — Venn diagram between the two replicates of the L. johnsonii La1 supernatant (Word). [file DataSheet3.DOCX]

**Venn diagram of identified proteins from *L. Johnsonii* La1 supernatant between the two biological replicates**

Venn diagram was realiszed using the Venny2.1 tool developped by Juan Carlos Oliveros (BioinfoGP, CNB-CSIC, Spain, <http://bioinfogp.cnb.csic.es/tools/venny/>)


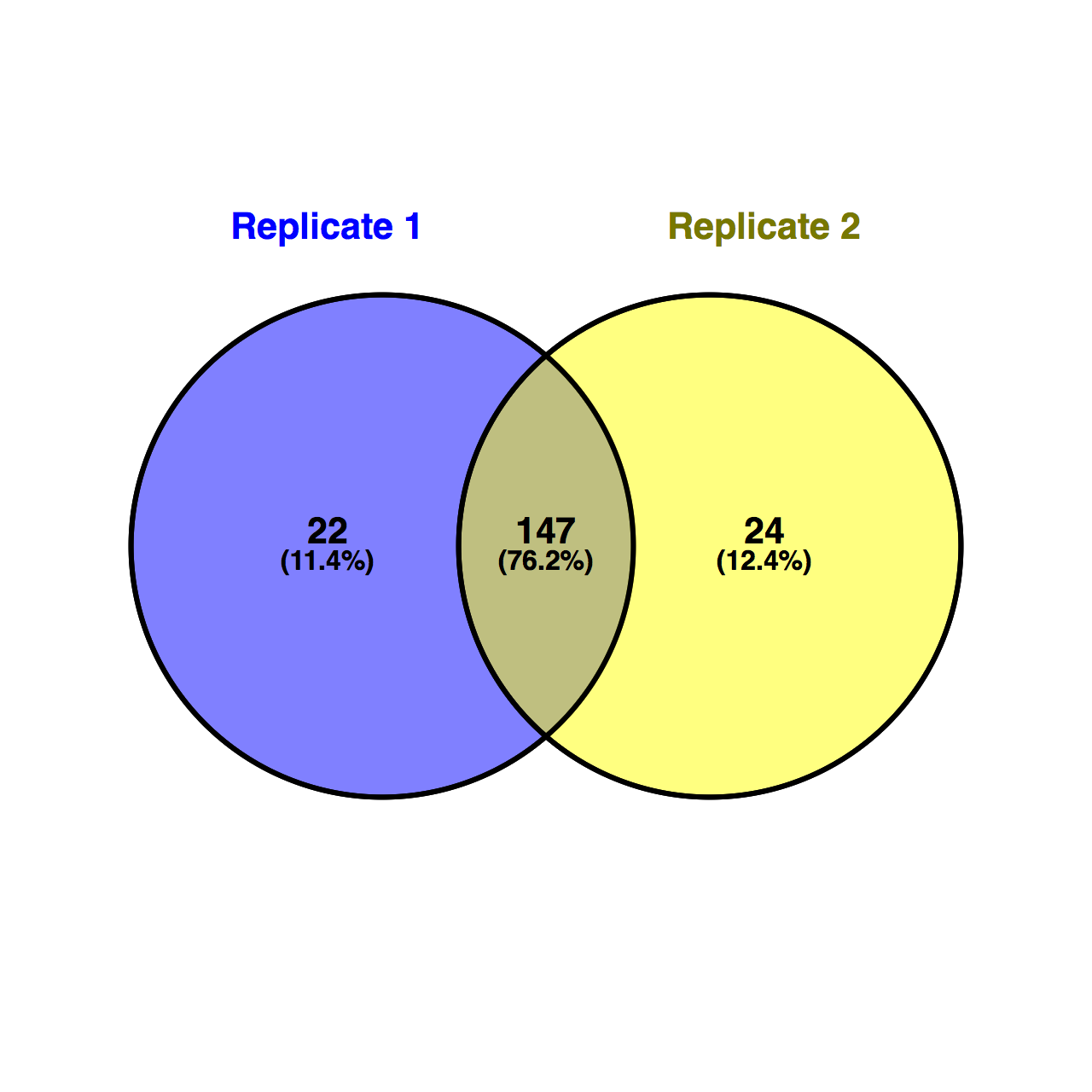


**22 elements included exclusively in "Replicate 1":**

41582817

41582856

41582962

41583137

41583366

41583574

41583778

41583847

41583933

41584180

262396998

262397001

262397120

262397146

262397648

262397838

262397995

262398134

262398406

262398441

262398570

262398604

**24 elements included exclusively in "Replicate 2":**

41582440

41582542

41582570

41582655

41582710

41582992

41582997

41583368

41583409

41583624

41583688

41583693

41583823

41583843

41583855

41584105

41584133

262397103

262397485

262397526

262398002

262398212

262398240

262398525

**147 common elements in "Replicate 1" and "Replicate 2":**

41582408

41582417

41582438

41582452

41582457

41582469

41582493

41582499

41582513

41582523

41582525

41582526

41582532

41582549

41582586

41582626

41582640

41582651

41582661

41582717

41582722

41582725

41582730

41582732

41582736

41582747

41582761

41582774

41582782

41582787

41582796

41582801

41582808

41582833

41582865

41582870

41582873

41582875

41582894

41582960

41583027

41583083

41583102

41583104

41583112

41583124

41583131

41583180

41583204

41583234

41583235

41583270

41583271

41583273

41583276

41583288

41583317

41583341

41583347

41583360

41583392

41583504

41583520

41583525

41583566

41583568

41583570

41583619

41583671

41583682

41583683

41583702

41583715

41583735

41583736

41583772

41583783

41583784

41583786

41583787

41583830

41583831

41583837

41583839

41583844

41583846

41583882

41583909

41583947

41583949

41583955

41583968

41584009

41584016

41584017

41584031

41584032

41584037

41584075

41584093

41584110

41584115

41584119

41584120

41584125

41584142

41584147

41584154

41584155

41584178

41584179

262396939

262396944

262397011

262397046

262397047

262397122

262397196

262397230

262397241

262397270

262397362

262397501

262397536

262397632

262397651

262397725

262397761

262397832

262397979

262398035

262398084

262398090

262398180

262398236

262398350

262398375

262398376

262398391

262398420

262398451

262398471

P02769

P00761

P02768-1

P04264

P35527
